# AI-Enabled Digital Twin Framework for TSCA-like Anomaly Detection in FPGA-SoC-Based Industrial Cyber-Physical Systems

**DOI:** 10.3390/s26144382

**Published:** 2026-07-10

**Authors:** Amrou Zyad Benelhaouare, Mohamed En-Nouar, Emmanuel Kengne, Ahmed Lakhssassi

**Affiliations:** Department of Engineering and Computer Science, University of Quebec in Outaouais, Gatineau, QC J9A 1L8, Canada; ennm05@uqo.ca (M.E.-N.); ekengne6@zjnu.edu.cn (E.K.); ahmed.lakhssassi@uqo.ca (A.L.)

**Keywords:** thermal side-channel attacks (TSCAs), thermal digital twin, FPGA-SoC, cyber-physical systems (CPSs), anomaly detection, machine learning, hardware security, industrial automation

## Abstract

Field-Programmable Gate Array System-on-Chip (FPGA-SoC) platforms are increasingly adopted in modern industrial Cyber-Physical Systems (CPSs), enabling real-time control, monitoring, and automation of critical industrial processes. The increasing integration density of modern FPGA-SoC architectures introduces new thermal security challenges, where heat evolves from a reliability concern into a potential source of information leakage. Thermal Side-Channel Attacks (TSCAs) exploit runtime thermal variations to infer sensitive operational, architectural, or cryptographic information from the underlying hardware. While this study is centered on FPGA-SoC platforms, comparable thermal security challenges are increasingly reported across other densely integrated computing architectures, including Multiprocessor System-on-Chip (MPSoC), System-in-Package (SiP), and emerging Three-Dimensional Integrated Circuit (3D-IC) technologies. Consequently, the detection of thermal side-channel intrusions has become a critical hardware security challenge for next generation industrial CPS infrastructures. To address this challenge, an AI-enabled Digital Twin (DT) framework is introduced for TSCA detection in densely integrated FPGA-SoC microarchitectures. By combining thermal behavioral modeling, feature engineering, and machine learning-based anomaly detection, the proposed framework extends conventional Thermal Digital Twin (TDT) approaches beyond monitoring and mitigation toward autonomous thermal threat detection. The proposed framework is experimentally validated using an NI myRIO-1900 platform integrating a Xilinx Zynq-7010 FPGA-SoC representative of modern industrial embedded control architectures. Experimental results demonstrate the feasibility of the proposed framework, achieving an accuracy of approximately 75% with an Area Under the ROC Curve (AUC) of 0.76 using a lightweight Isolation Forest model. These results validate the capability of the proposed AI-enabled Digital Twin framework to learn normal thermal behavioral patterns and autonomously detect anomalous thermal activities potentially related to TSCAs.

## 1. Introduction

Industrial Cyber-Physical Systems (CPSs) have emerged as a fundamental technological pillar of Industry 4.0, enabling the integration of computation, communication, sensing, and control within modern manufacturing environments [1,2]. These systems support a wide range of mission critical applications, including industrial automation, intelligent monitoring, predictive maintenance, machine control, and real-time decision-making [3]. To meet increasingly stringent requirements in terms of latency, determinism, reliability, and computational performance, industrial control platforms are progressively adopting FPGA-based embedded architectures capable of combining software programmability with hardware acceleration [4].

**Figure 1:**
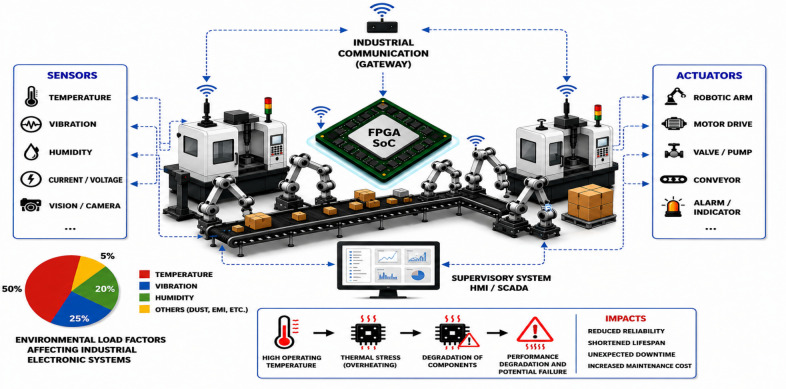
Central role of FPGA-SoC platforms in industrial Cyber-Physical Systems (CPS), adapted from [8].

Recent studies identify Field-Programmable Gate Arrays (FPGAs) as a key enabling technology for next generation industrial control systems due to their reconfigurability, parallel processing capabilities, and ability to execute time critical tasks with deterministic behavior [5]. Representative industrial platforms such as National Instruments CompactRIO and myRIO integrate FPGA-SoC technologies to support real-time monitoring [6], control, and Industrial Internet of Things (IIoT) applications. FPGA-SoC platforms are expected to play a growing role in future intelligent manufacturing and industrial CPS ecosystems [7]. As illustrated in Figure 1, FPGA-SoC platforms occupy a central position within industrial CPS architectures. Beyond enabling real-time control and communication, their continuous operation under demanding workloads may expose them to significant thermal stress, making thermal behavior an increasingly important concern for system reliability and security.

The increasing adoption of FPGA-SoC platforms in industrial CPS has been accompanied by a continuous growth in integration density. Modern FPGA-SoC devices integrate processors, programmable logic, memory resources, and communication interfaces within a single chip, resulting in increasingly complex thermal behaviors during operation [9]. As a consequence, temperature variations generated by hardware activity become more pronounced and observable, particularly under dynamic workloads and real-time control tasks [10]. While thermal effects have traditionally been investigated from reliability and thermal management perspectives, recent studies have demonstrated that thermal emissions may also reveal information about the underlying execution behavior of a computing system [11,12]. This observation has raised growing concerns regarding the security implications of thermal information leakage in densely integrated FPGA-SoC architectures, where thermal side channels may expose sensitive operational or architectural information to potential adversaries [13].

Thermal information leakage has given rise to a new class of hardware security threats known as Thermal Side-Channel Attacks (TSCAs) [12]. These attacks exploit temperature variations generated during system operation to infer sensitive information about the underlying hardware activity. Recent studies have demonstrated that thermal signatures can reveal execution patterns [14], resource utilization behaviors, and even security critical operations through the observation of heat propagation effects. In densely integrated FPGA-SoC architectures, where heterogeneous computing resources coexist on the same chip, thermal interactions become more pronounced, increasing the risk of information leakage [15]. As these platforms are widely deployed in industrial Cyber-Physical Systems (CPS), the exploitation of thermal side channels may compromise sensitive operational information and threaten the security of critical industrial processes.

To address the growing cybersecurity challenges in industrial Cyber-Physical Systems (CPS), recent research has begun exploring Digital Twin (DT) technologies beyond their traditional roles in system monitoring, simulation, and predictive maintenance. Several studies have demonstrated that DTs can serve as effective platforms for security monitoring and anomaly detection by continuously mirroring the behavior of physical industrial systems. For instance, Akbarian et al. integrated intrusion detection mechanisms within industrial Digital Twins to identify cyberattacks without affecting live system operations [16]. Similarly, Varghese et al. proposed a DT-based intrusion detection framework for Industrial Control Systems (ICS), combining attack simulation capabilities with machine learning techniques for near real-time attack detection and classification [17]. More recently, Lucchese et al. employed Digital Twins and process mining techniques to uncover anomalous behaviors and cyberattacks in industrial environments [18]. These studies collectively demonstrate the growing potential of DT technologies as cybersecurity enablers capable of supporting anomaly detection, threat monitoring, and security assessment in complex industrial systems.

However, despite these promising advances, the application of Digital Twin technologies to thermal side-channel security remains largely unexplored. Existing DT-based security frameworks mainly focus on network intrusions, cyberattacks, or process anomalies, while thermal information leakage and Thermal Side-Channel Attacks (TSCAs) have received limited attention. Furthermore, current Thermal Digital Twin (TDT) approaches primarily concentrate on thermal monitoring, prediction, and management objectives, without incorporating intelligent learning mechanisms capable of autonomously distinguishing legitimate thermal behaviors from adversarial thermal signatures [19,20]. This limitation motivates the development of the AI-Enabled Digital Twin framework proposed in this work.

The main contributions of this paper are summarized as follows:

- Develop a thermal feature engineering pipeline for FPGA-SoC behavioral characterization.
- Propose an Isolation Forest-based mechanism for TSCA-like anomaly detection.
- Design an AI-enabled Thermal Digital Twin for offline anomaly analysis.
- Validate the framework experimentally on an NI myRIO-1900 (National Instruments Corporation, Austin, TX, USA) platform.
- Introduce a future Edge-AI pathway toward online, real-time, and on-device Digital Twin deployment.

The remainder of this paper is organized as follows. Section 2 presents the proposed AI-Enabled Digital Twin framework through an industrial FPGA-SoC case study. Section 3 describes the acquisition of raw thermal signatures and the construction of the thermal behavioral dataset through feature engineering techniques. Section 4 introduces the machine learning-based TSCA detection methodology using the Isolation Forest algorithm. Section 5 discusses the experimental results obtained from the proposed framework and outlines future research directions. Section 6 concludes the paper and summarizes the main findings of this work.

## 2. AI-Enabled Digital Twin Framework and Industrial FPGA-SoC Case Study

### 2.1. Industrial FPGA-SoC Case Study

To validate the proposed AI-Enabled Thermal Digital Twin framework, an experimental platform based on the National Instruments (NI) myRIO-1900 was developed. The myRIO-1900 integrates a Xilinx Zynq-7010 SoC (Advanced Micro Devices, Inc., Santa Clara, CA, USA), combining a dual-core ARM Cortex-A9 processing system operating at 667 MHz with FPGA programmable logic resources within a single device [21]. The Zynq-7010 contains approximately 28K logic cells, 80 DSP slices, 240 KB of block RAM, and heterogeneous processing resources that enable the implementation of real-time embedded control applications. An important feature of the selected FPGA-SoC platform is the presence of an integrated on-chip thermal sensing infrastructure accessible through the Xilinx System Monitor (SYSMON/XADC) [22]. This thermal monitoring capability is essential for real-time supervision of the device’s operating conditions during embedded applications.

### 2.2. Technical Specifications

The temperature sensor integrated into the Xilinx Zynq-7010 FPGA-SoC presents the following characteristics [22]:

- Temperature measurement accuracy: ±4 °C for nominal operating conditions (−40 °C ≤ *T_j_* ≤ 100 °C).
- Extended temperature measurement accuracy: ±6 °C for extended operating conditions (−55 °C ≤ *T_j_* < −40 °C or 100 °C < *T_j_* ≤ 125 °C).

### 2.3. XADC Architecture

The XADC block within the Zynq-7010 contains two 12-bit analog-to-digital converters (ADCs) operating at 1 MSPS (million samples per second). Beyond the dedicated temperature sensors, the XADC integrates multiple on-chip sensing capabilities: thermal sensors for junction temperature monitoring and supply voltage sensors for power rail supervision. These sensors enable continuous monitoring through the JTAG interface, allowing real-time acquisition of on-die temperature and voltage measurements directly from the silicon substrate.

Although the XADC provides direct on-chip thermal monitoring with a 12-bit conversion resolution and nominal accuracy of ±4 °C under standard operating conditions, minor measurement uncertainty may still arise from ADC quantization effects and ambient thermal fluctuations. Nevertheless, its sensing precision remains sufficiently reliable for relative thermal pattern analysis and anomaly-oriented thermal profiling.

### 2.4. Practical Applications

The integrated thermal sensor facilitates dynamic thermal management in the myRIO-1900 platform, enabling developers to implement temperature aware control algorithms and adaptive power management strategies. This on-chip sensing capability eliminates the need for external temperature measurement circuits while providing direct access to the SoC’s instantaneous thermal state.

**Figure 2:**
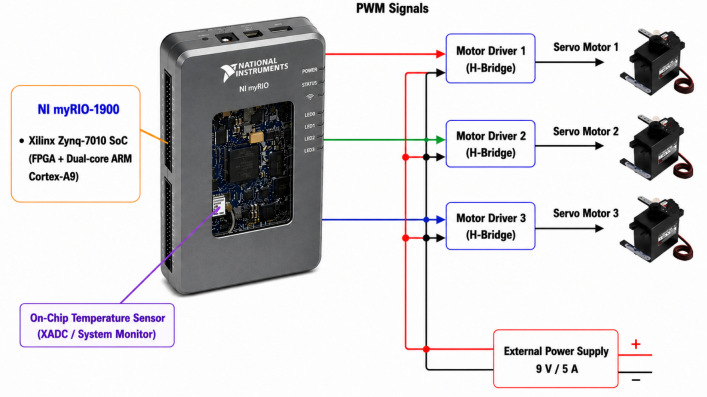
Experimental setup of the NI myRIO-1900 FPGA-SoC and three-servo-motor control system.

As illustrated in Figure 2, the physical process considered in this work consists of three independently controlled servo motors connected to the FPGA-SoC platform. Each motor is driven through dedicated PWM control signals generated by the programmable logic. The use of multiple servo actuators enables the generation of different operating workloads through various activation combinations, including idle operation, single-motor activity, dual-motor activity, and simultaneous activation of all three motors. These operating conditions induce distinct computational and thermal behaviors within the FPGA-SoC, which are subsequently captured through the on-chip thermal sensing infrastructure.

Although simplified, the proposed setup reproduces the fundamental architecture of an industrial Cyber-Physical System (CPS), where a central embedded controller continuously acquires information, executes control algorithms, and coordinates physical actuators in real time. Similar FPGA-SoC-based architectures are commonly encountered in industrial automation systems, robotic manipulators, motion-control platforms, automated production lines, and intelligent manufacturing environments. In this context, the NI myRIO-1900 acts as the control and command unit, while the servo motors emulate industrial actuation devices interacting with the physical environment.

The combination of real-time actuation, embedded processing, FPGA programmability, and on-chip thermal sensing makes this platform particularly suitable for investigating thermal behavioral signatures and evaluating the proposed AI-Enabled Thermal Digital Twin framework for Thermal Side-Channel Attack (TSCA) detection.

### 2.5. Operational Scenarios

To investigate the thermal behavior of the considered FPGA-SoC platform under different operating conditions, a set of representative workload scenarios was defined. The NI myRIO-1900 controls three servo motors acting as industrial actuators, while the integrated on-chip thermal sensor continuously monitors the junction temperature of the Xilinx Zynq-7010 device. The selected scenarios emulate different levels of actuation activity commonly encountered in industrial Cyber-Physical Systems (CPS), thereby generating distinct thermal signatures within the FPGA-SoC.

Four baseline operating conditions were considered according to the number of active servo motors:

- S0: Baseline operating condition (no active servo motor);
- S1: Single-actuator operating condition (one active servo motor);
- S2: Dual-actuator operating condition (two active servo motors operating simultaneously);
- S3: Triple-actuator operating condition (three active servo motors operating simultaneously).

These scenarios produce increasing computational and control workloads within the FPGA-SoC due to variations in PWM generation, I/O activity, and embedded control operations. Consequently, each operating condition generates a characteristic thermal behavior that can be captured through the integrated thermal sensing infrastructure.

All operational scenarios were executed under controlled laboratory conditions (ambient temperature maintained at approximately 24 ± 1 °C) to ensure metrological consistency. For experimental repeatability and reproducibility, each thermal scenario was repeated multiple times using identical FPGA configurations, fixed PWM control parameters, unchanged actuator sequences, and a constant thermal sampling interval. The resulting thermal traces showed only minor variations across successive acquisitions, and the final dataset was constructed from the averaged thermal behavior to strengthen measurement reliability and experimental validation.

To emulate abnormal thermal behavior potentially associated with Thermal Side-Channel Attacks (TSCAs), a TSCA-like workload was introduced in parallel with each baseline operating condition. The TSCA-like activity consists of an additional software-generated computational load executed on the FPGA-SoC without altering the normal motor control functionality. This supplementary workload increases the internal switching activity and processing demand of the device, producing subtle thermal disturbances observable through the on-chip temperature sensor.

Although simplified, this TSCA-like workload emulates the localized computational stress and heat propagation patterns commonly reported in practical thermal side-channel leakage scenarios.

Accordingly, each baseline scenario was paired with its corresponding anomalous counterpart:

- S0-N: Baseline operating condition;
- S0-A: Baseline operating condition with TSCA-like workload;
- S1-N: Single-actuator operating condition;
- S1-A: Single-actuator operating condition with TSCA-like workload;
- S2-N: Dual-actuator operating condition;
- S2-A: Dual-actuator operating condition with TSCA-like workload;
- S3-N: Triple-actuator operating condition;
- S3-A: Triple-actuator operating condition with TSCA-like workload.

The objective of these scenarios is to evaluate the capability of the proposed AI-Enabled Thermal Digital Twin framework to distinguish normal thermal behaviors from anomalous thermal deviations under varying operating conditions. Rather than reproducing a specific attack implementation, the TSCA-like workload serves as a controlled source of thermal disturbance that mimics the additional activity potentially generated during side-channel exploitation attempts. This approach provides a practical and reproducible environment for validating the proposed thermal anomaly detection methodology.

For clarity, Table 1 summarizes the complete set of baseline and TSCA-like operating scenarios considered throughout this study.

### 2.6. AI-Enabled Digital Twin Framework

**Figure 3:**
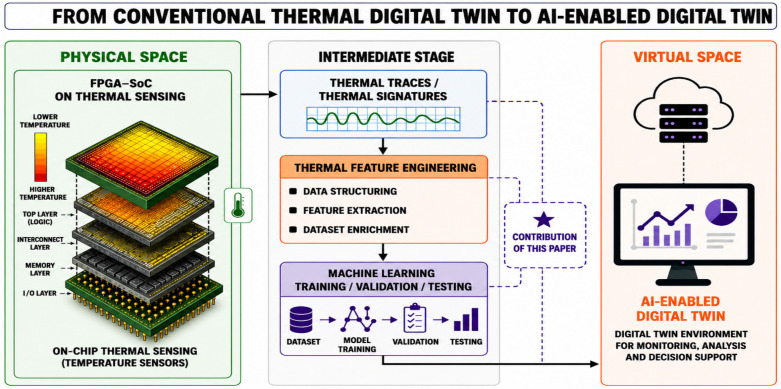
Conceptual architecture of the proposed AI-Enabled Thermal Digital Twin framework for TSCA detection.

The proposed AI-Enabled Thermal Digital Twin framework and its main building blocks are illustrated in Figure 3. The framework establishes a bridge between the physical thermal sensing layer and the virtual intelligence layer through thermal behavioral feature engineering and machine learning driven anomaly detection.

The framework is composed of three interconnected layers. The Physical Space corresponds to the FPGA-SoC platform where thermal information is acquired through the integrated on-chip temperature sensor. The Intermediate Stage transforms raw thermal measurements into representative thermal behavioral signatures through feature engineering and machine learning preparation processes. Finally, the Virtual Space hosts the intelligent Digital Twin model responsible for anomaly detection and thermal security monitoring.

Unlike conventional Thermal Digital Twins that mainly provide thermal visualization, monitoring, and state representation functionalities, the proposed framework extends the Digital Twin concept by incorporating machine learning-based intelligence. This additional capability enables autonomous interpretation of thermal behaviors and supports the identification of abnormal thermal patterns potentially associated with Thermal Side-Channel Attacks (TSCAs).

At the current stage, the proposed AI-Enabled Thermal Digital Twin is implemented as an offline analytical framework, where thermal data are acquired, processed, and analyzed after experimentation. Real-time and closed-loop deployment for embedded autonomous thermal monitoring remains part of future work.

The following section focuses on the acquisition of raw thermal signatures and the construction of thermal behavioral datasets required for the development of the proposed AI-Enabled Thermal Digital Twin.

## 3. Thermal Signatures and Dataset Construction

### 3.1. Software Development Environment

**Figure 4:**
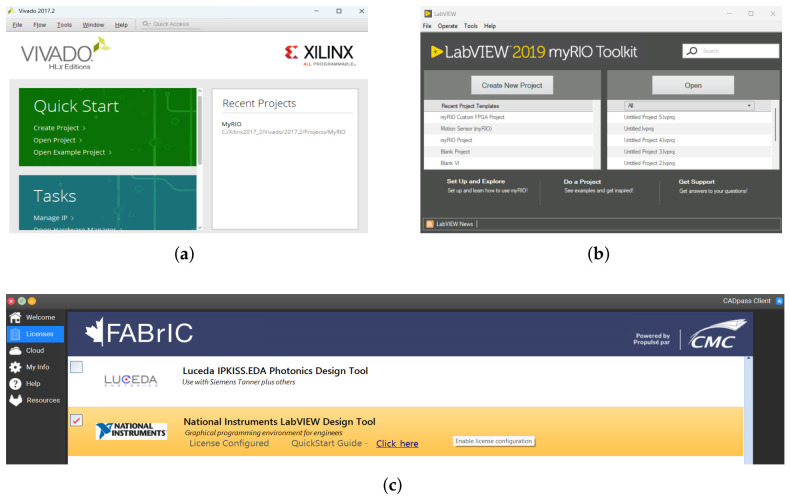
Software development environment used for FPGA design, XADC configuration, and real-time thermal data acquisition. (**a**) Xilinx Vivado Design Suite 2017.2. (**b**) NI LabVIEW FPGA 2019 with myRIO Toolkit. (**c**) CMC Microsystems FABrIC Academic Licensing Platform for Xilinx and National Instruments Tools.

The experimental framework relies on a hardware, software co-design environment combining Xilinx Vivado Design Suite 2017.2 and National Instruments LabVIEW FPGA 2019 with the myRIO Toolkit. This software combination is fully compatible with the NI myRIO-1900 platform integrating a Xilinx Zynq-7010 FPGA-SoC and enables seamless interaction between FPGA hardware resources and high-level graphical programming environments (Figure 4).

Access to the Xilinx, AMD and National Instruments development environments was provided through the CMC Microsystems FABrIC research infrastructure (Figure 4c). These licenses are granted to the Laboratoire d’Ingenierie et de Microsystemes avancés (LIMA), Université du Québec en Outaouais (UQO), through a research agreement supporting advanced FPGA design, embedded systems development, and cyber-physical systems research.

Vivado 2017.2 (Figure 4a) was used to configure and synthesize the XADC (System Monitor) IP core responsible for on-chip temperature sensing. After hardware generation, the custom FPGA design was exported and integrated into the LabVIEW FPGA environment (Figure 4b), allowing direct access to the XADC temperature outputs from user-developed Virtual Instruments (VIs).

This co-design workflow enables real-time acquisition of FPGA junction temperature measurements without requiring external sensing circuitry. Through dedicated LabVIEW FPGA VIs, thermal data can be continuously monitored, processed, and recorded during system operation, thereby providing the thermal traces required for the construction of the proposed AI-Enabled Thermal Digital Twin framework.

Although newer releases of both Xilinx Vivado and National Instruments LabVIEW are available, Vivado 2017.2 and LabVIEW FPGA 2019 were intentionally selected in this work due to their proven compatibility with the NI myRIO-1900 platform and its embedded Xilinx Zynq-7010 FPGA-SoC. In particular, this software combination provides stable support for the integration and deployment of custom XADC-based thermal sensing functionalities within the LabVIEW FPGA environment. Preliminary investigations using more recent software versions revealed increased integration complexity and limited accessibility to low-level XADC resources required for direct thermal monitoring. Therefore, the selected toolchain offers a reliable and well-documented framework for configuring the XADC IP core, importing FPGA designs into LabVIEW FPGA, and performing real-time on-chip temperature acquisition throughout the experimental campaign.

### 3.2. XADC Configuration and Thermal Sensor Activation

#### 3.2.1. XADC-Based On-Chip Temperature Monitoring Setup

**Figure 5:**
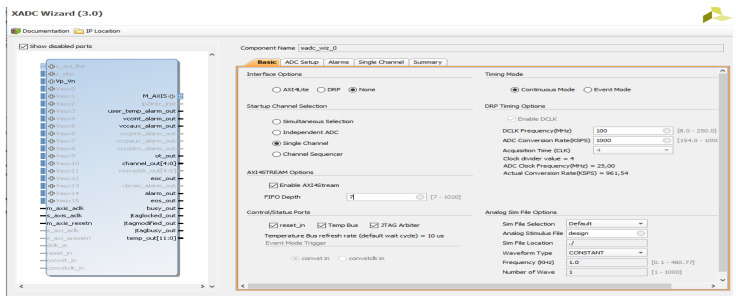
XADC Configuration.

The thermal acquisition infrastructure relies on the Xilinx System Monitor (XADC), which provides direct access to the integrated temperature sensing circuitry embedded within the Zynq-7010 FPGA-SoC. The XADC IP core was configured in Vivado 2017.2 using the XADC Wizard, as illustrated in Figure 5.

For this work, the temperature sensor channel was enabled in continuous acquisition mode to ensure uninterrupted monitoring of the FPGA junction temperature. The internal temperature bus output (temp_out) was activated and exported as a digital signal accessible from the FPGA fabric. Continuous conversion mode was selected to provide periodic temperature updates without requiring external triggering mechanisms.

The resulting configuration allows the thermal sensing subsystem to operate autonomously and deliver real-time temperature measurements throughout all experimental scenarios.

#### 3.2.2. FPGA Integration of the Thermal Monitoring Core

**Figure 6:**
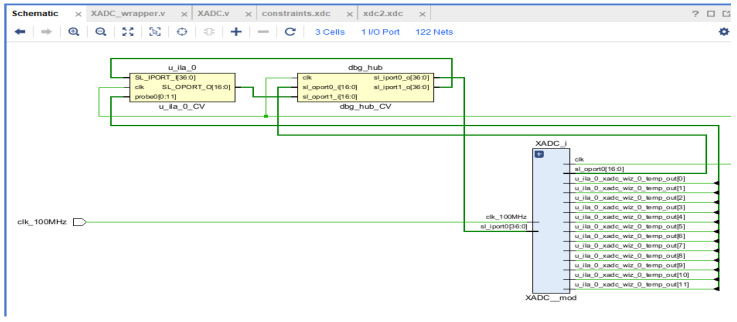
XADC IP Integration.

After configuration, the XADC IP core was integrated into the FPGA design as shown in Figure 6. The thermal monitoring module receives the FPGA clock signal and continuously generates digital temperature samples through the (temp_out) interface. To facilitate debugging and validation, the temperature output bus was connected to the Integrated Logic Analyzer (ILA), allowing direct observation of thermal measurements inside the FPGA fabric. This architecture enables hardware-level verification of the thermal sensing process before exporting the temperature data to the LabVIEW environment.

The FPGA implementation therefore establishes the first layer of the proposed digital twin infrastructure by providing direct access to on-chip thermal information.

#### 3.2.3. Hardware-Level Thermal Validation

**Figure 7:**
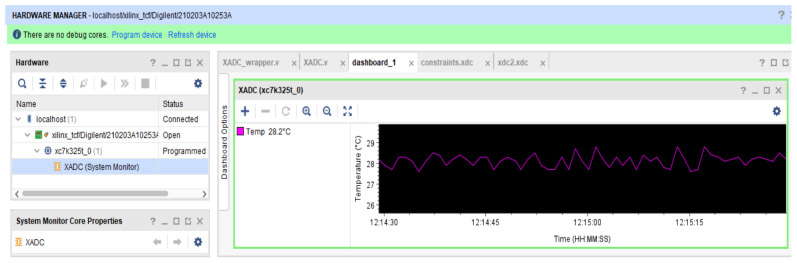
XADC Monitoring.

Prior to large-scale thermal data acquisition, the functionality of the XADC subsystem was validated using the Vivado Hardware Manager and the integrated System Monitor interface, shown in Figure 7.

The System Monitor provides direct visualization of the FPGA junction temperature and internal supply voltages in real time. Successful observation of stable temperature traces confirmed the correct operation of the XADC sensor and the integrity of the thermal measurement chain.

This validation step ensured that the generated thermal data originated directly from the FPGA silicon substrate rather than from external sensing devices.

#### 3.2.4. Integration of the XADC IP Core into the LabVIEW FPGA Environment

Although the XADC temperature sensor is physically embedded inside the Zynq-7010 FPGA-SoC, accessing its measurements from the NI myRIO-1900 platform requires a complete integration workflow between the Xilinx Vivado design environment and the LabVIEW FPGA framework.

**Figure 8:**
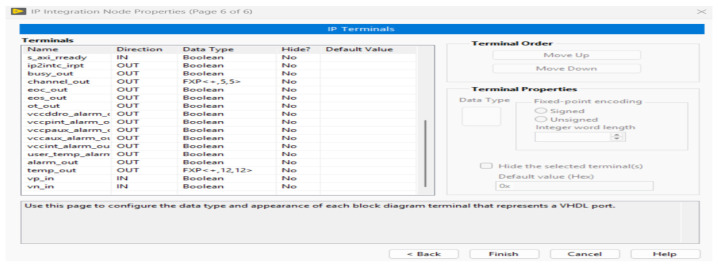
Importation of the XADC IP core into the LabVIEW FPGA environment through the IP Integration Node interface.

The XADC IP core was first configured in Vivado 2017.2 and exported as a reusable hardware module. The generated IP was subsequently imported into LabVIEW FPGA through the IP Integration Node interface, allowing direct access to the temperature output bus (temp_out) from the graphical programming environment, as illustrated in Figure 8. This step establishes the communication bridge between the Xilinx-generated hardware description and the LabVIEW FPGA design flow.

**Figure 9:**
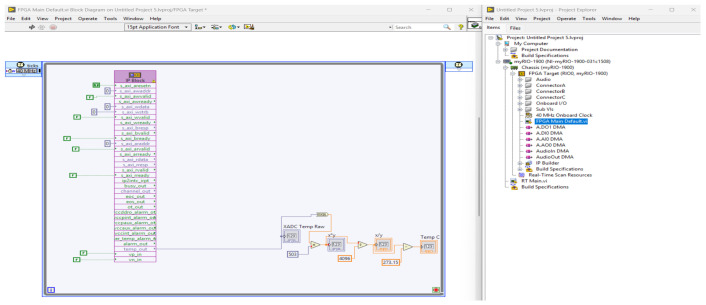
LabVIEW FPGA block diagram for XADC.

After successful importation, the XADC module was instantiated inside the FPGA Main VI block diagram, where the raw thermal measurements were processed and converted into physical temperature values expressed in degrees Celsius. Figure 9 shows the FPGA-level implementation, including the temperature conversion logic and the data path used to access the thermal sensor output. This implementation enables direct acquisition of junction temperature measurements from the silicon substrate in real time.

**Figure 10:**
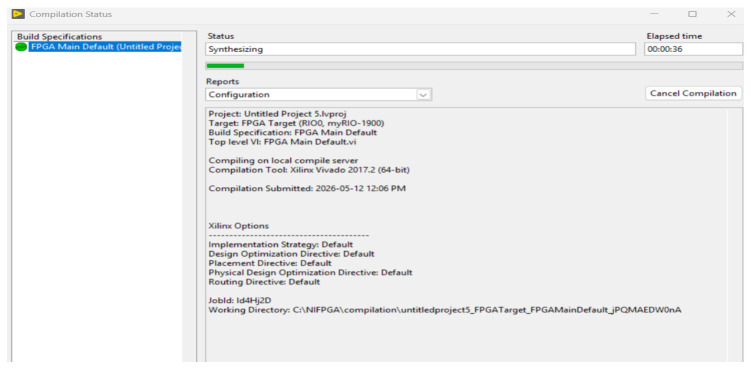
LabVIEW FPGA compilation workflow invoking Xilinx Vivado 2017.2 synthesis and bitstream generation for the NI myRIO-1900 platform.

Once the FPGA application was completed, the design was compiled using the LabVIEW FPGA compilation workflow. As shown in Figure 10, LabVIEW automatically invokes the Xilinx Vivado 2017.2 synthesis and implementation tools to generate the FPGA bitstream. During this process, the imported XADC IP core is synthesized, placed, routed, and integrated into the final hardware configuration targeting the Zynq-7010 device embedded within the NI myRIO-1900 platform.

Following successful compilation, the generated bitstream is deployed to the FPGA fabric of the myRIO device, enabling continuous thermal monitoring during runtime. This workflow provides seamless access to the embedded thermal sensing infrastructure while maintaining compatibility between the NI LabVIEW ecosystem and the Xilinx FPGA development tools.

### 3.3. Raw Thermal Dataset and Thermal Signatures

**Figure 11:**
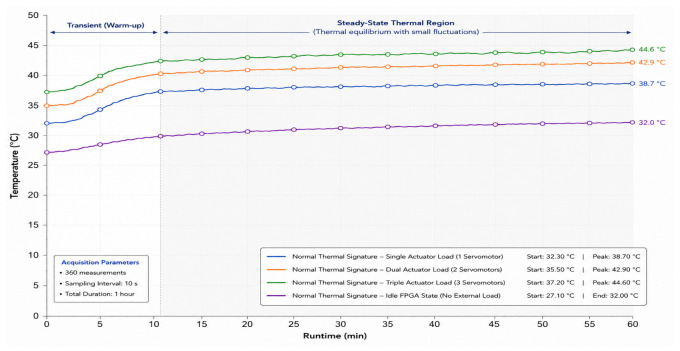
Raw thermal signatures acquired during one hour for the four normal operating scenarios.

The thermal acquisition campaign produced a set of raw thermal signatures corresponding to the operating scenarios previously defined in Section 2.5. Figure 11 presents the temperature evolution recorded during one hour for the four normal operating conditions. The obtained thermal traces exhibit a consistent thermal behavior characterized by an initial transient heating phase followed by a steady-state region.

As expected, increasing the actuator activity leads to higher equilibrium temperatures. The Idle FPGA scenario remains the coolest operating condition, while the 3 Servomotors scenario produces the highest thermal footprint. The thermal separation observed between the four signatures confirms that the selected scenarios generate distinguishable thermal behaviors suitable for subsequent feature extraction and machine learning analysis.

To evaluate the capability of the proposed framework to distinguish abnormal thermal behaviors, an additional TSCA-like condition was introduced. The abnormal thermal load was activated during runtime while maintaining the same external operating conditions associated with each scenario.

**Figure 12:**
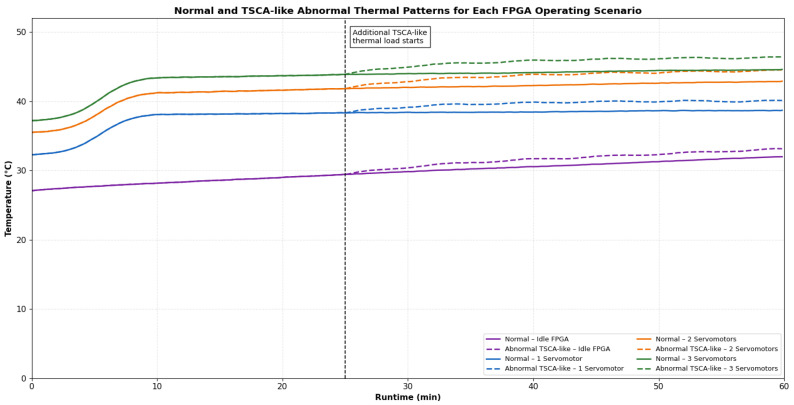
Normal and TSCA-like abnormal thermal signatures for each FPGA operating scenarios.

Figure 12 compares the normal and TSCA-like thermal signatures. A clear thermal divergence can be observed after the activation instant, resulting in a measurable temperature elevation across all operating conditions. Although the thermal increase remains moderate, the resulting patterns exhibit a persistent deviation from their nominal counterparts, creating a representative dataset for anomaly detection and AI-enabled digital twin validation.

### 3.4. Thermal Feature Engineering for AI-Enabled Digital Twin

Although the raw thermal signatures provide valuable information about the FPGA operating conditions, directly using time-series measurements as inputs for machine learning models is generally inefficient and computationally expensive. Therefore, a feature engineering stage was introduced to transform the acquired thermal traces into a compact and informative representation suitable for AI-enabled Digital Twin applications.

The objective of this process is to extract discriminative thermal descriptors capable of characterizing both the global thermal behavior and the local temporal dynamics of each operating scenario. Four complementary categories of features were considered.

#### 3.4.1. Differential Thermal Features

The first category consists of differential thermal features computed from the successive temperature increments:

ΔT(i)=T(i)−T(i−1)

**Figure 13:**
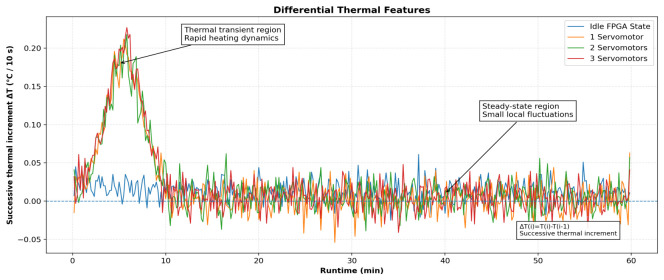
Differential thermal features extracted from the raw thermal signatures.

These features capture the instantaneous heating dynamics of the FPGA. As illustrated in Figure 13, the warm-up phase is associated with large positive thermal increments, whereas the steady-state region exhibits only small local fluctuations around zero.

This representation highlights the thermal transient characteristics that may not be directly observable from the original temperature signal.

#### 3.4.2. Global Statistical Features

The second category summarizes each thermal signature through global statistical descriptors, including minimum temperature, maximum temperature, mean temperature, and thermal variability. These features provide a compact representation of the overall thermal behavior associated with each operating scenario.

**Figure 14:**
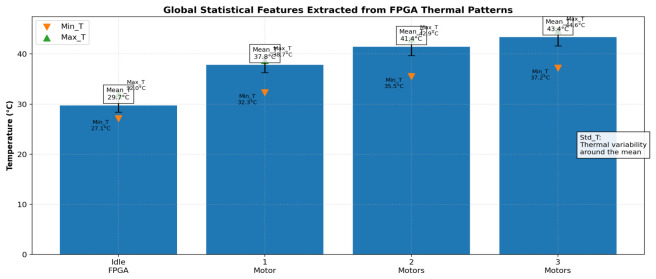
Global statistical features extracted from raw thermal signatures.

As shown in Figure 14, the extracted statistics preserve the thermal hierarchy observed during the experiments, where higher actuator activity results in higher average and peak temperatures.

#### 3.4.3. Window-Based Mean Features

**Figure 15:**
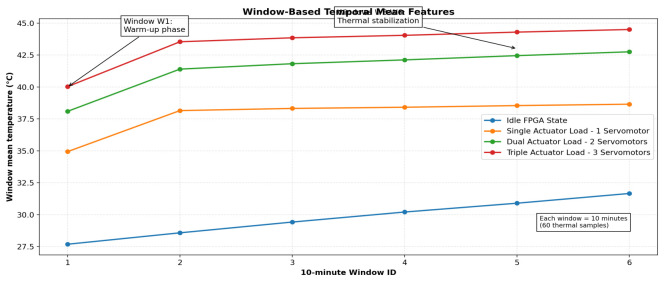
Window-based mean thermal features.

To preserve temporal information while reducing the dimensionality of the dataset, the one-hour thermal traces were divided into six consecutive windows of ten minutes each. The average temperature was then computed for every window (Figure 15).

Figure 15 shows that this approach effectively captures the transition from the initial heating phase toward thermal stabilization while preserving the thermal distinction between the operating scenarios.

#### 3.4.4. Thermal Slope Analysis Using Sliding Windows

Finally, window-based thermal slopes were extracted to quantify the heat propagation rate during each acquisition interval. These features represent the temporal derivative of the thermal evolution and provide information about the heating tendency of the system.

**Figure 16:**
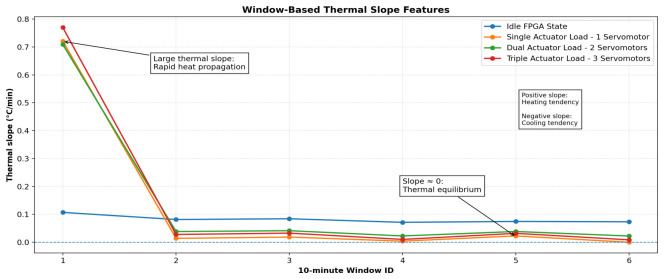
Window-based thermal slope features.

As depicted in Figure 16, the largest slopes occur during the initial thermal transient, whereas values close to zero indicate that the FPGA has reached thermal equilibrium. Such dynamic descriptors are particularly relevant for anomaly detection and Digital Twin predictive models.

The thermal acquisition and data engineering process resulted in a structured dataset combining raw thermal signatures and engineered features extracted from the different FPGA operating scenarios. This dataset constitutes the training foundation of the proposed AI-enabled Digital Twin and supports the Isolation Forest learning model adopted in this work.

Table 2 summarizes the structure of the constructed thermal dataset and the engineered thermal features used for the machine learning-based anomaly detection stage.

The next section introduces the machine learning framework developed for thermal state classification and prediction.

## 4. Machine Learning Model Development and Validation

### 4.1. Isolation Forest Performance

The proposed AI-enabled Digital Twin integrates an Isolation Forest model trained under an unsupervised anomaly detection protocol. The engineered dataset contains 536 thermal windows, including 268 normal windows and 268 TSCA-like anomalous windows. Each window is described by 17 thermal features extracted from the raw temperature traces.

Among unsupervised anomaly detection methods, Isolation Forest was selected due to its computational efficiency, low training complexity, and robustness when handling low-dimensional statistical feature spaces. Compared to One-Class SVM, which often requires careful kernel tuning, and autoencoders, which typically demand larger datasets and higher training costs, Isolation Forest provides a lightweight and scalable solution suitable for the present thermal anomaly detection framework.

Unlike a conventional supervised train/test split, the Isolation Forest model was trained exclusively on the normal thermal subset composed of 268 samples. The complete dataset of 536 samples was then used for evaluation, including both normal and anomalous thermal windows. This protocol reflects the intended anomaly detection use case, where only normal behavior is assumed to be available during training.

A contamination factor of 0.25 was selected after sensitivity analysis, providing the best overall performance. The final model achieved an Accuracy of 75.2%, with a Precision of 75.1%, a Recall of 75.4%, and an F1-score of 75.2%. The close values of these metrics indicate a balanced behavior between anomaly detection capability and false alarm control.

### 4.2. Confusion Matrix Analysis

**Figure 17:**
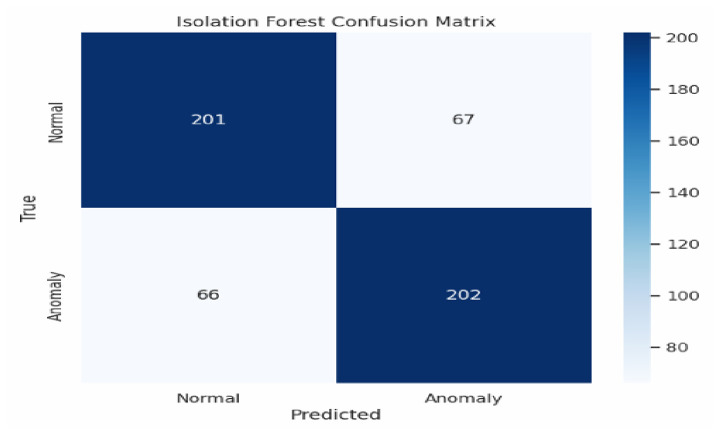
Confusion matrix.

Figure 17 presents the confusion matrix of the proposed model. The Isolation Forest correctly identified 201 normal windows and 202 anomalous windows, while only 67 false positives and 66 false negatives were produced.

From the confusion matrix, the False Positive Rate (FPR) and False Negative Rate (FNR) were computed as follows:

$$\mathrm{FPR}=\frac{FP}{FP+TN}=\frac{67}{67+201}=25.0\%$$

$$\mathrm{FNR}=\frac{FN}{FN+TP}=\frac{66}{66+202}=24.6\%$$

The obtained FPR and FNR values indicate a balanced trade-off between false alarms and missed anomaly detections.

These results demonstrate that the thermal features extracted from the FPGA are sufficiently discriminative to separate normal and abnormal operating conditions with a balanced detection capability.

### 4.3. Distribution Analysis of Anomaly Scores

**Figure 18:**
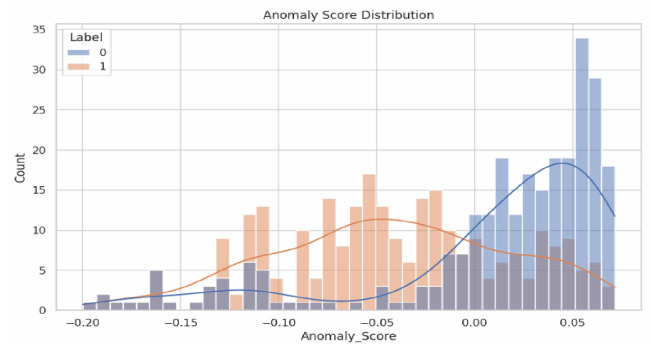
Anomaly score distribution.

Figure 18 illustrates the distribution of anomaly scores generated by the Isolation Forest. Normal thermal behaviors are mainly associated with higher scores, whereas anomalous conditions tend to produce lower scores.

Although a partial overlap exists between the two distributions, a clear tendency toward separation can be observed, confirming that abnormal thermal activities generate measurable deviations in the extracted thermal signatures.

### 4.4. Principal Component Analysis of Thermal Features

**Figure 19:**
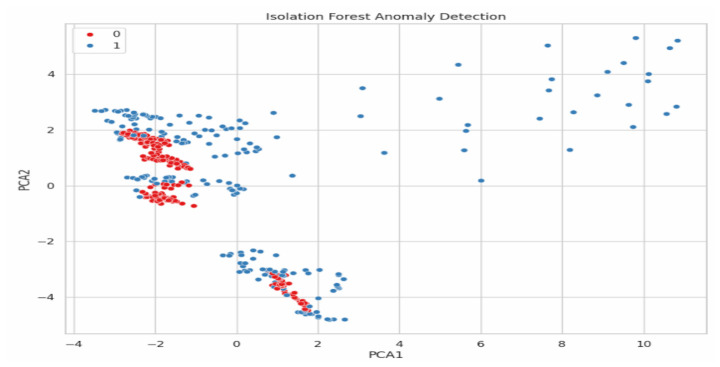
PCA-Based Visualization.

To better understand the model behavior, the extracted thermal features were projected into a two-dimensional Principal Component Analysis (PCA) space, as shown in Figure 19.

The visualization reveals that a significant portion of anomalous thermal windows occupies regions different from normal operating conditions. This confirms that the engineered thermal features preserve relevant information for unsupervised anomaly detection.

### 4.5. ROC Analysis

**Figure 20:**
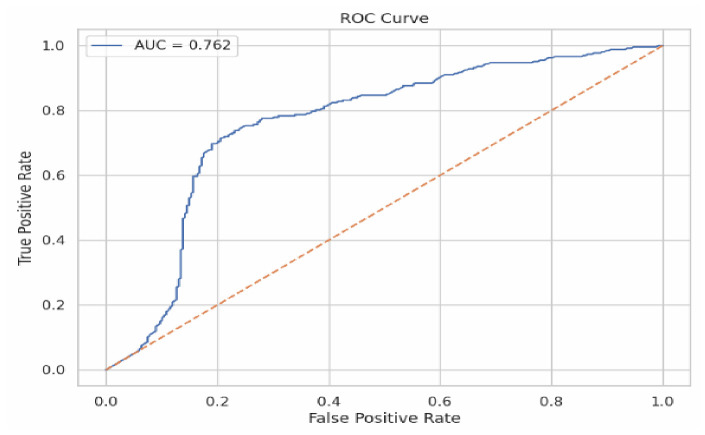
Receiver Operating Characteristic (ROC) curve performance.

The Receiver Operating Characteristic (ROC) curve, shown in Figure 20, achieved an Area Under the Curve (AUC) of 0.762.

To synthesize the quantitative evaluation of the proposed Isolation Forest-based anomaly detection model, Table 3 summarizes the principal classification and discrimination metrics obtained across the complete thermal dataset.

This result indicates that the proposed AI-enabled Digital Twin possesses a good capability to discriminate between normal and anomalous FPGA thermal behaviors, validating the effectiveness of the selected thermal features for unsupervised monitoring.

Overall, the experimental results confirm that thermal statistical features extracted from the FPGA provide sufficient information for anomaly detection without requiring labeled anomalous data during training. The obtained performance supports the feasibility of integrating an Isolation Forest into an AI-enabled Digital Twin framework for FPGA thermal monitoring and early detection of abnormal operating conditions.

## 5. Discussion of Results & Future Research Directions

The results presented throughout this work demonstrate the feasibility of constructing an AI-enabled Digital Twin for FPGA thermal monitoring based on real-time thermal acquisition and machine learning techniques. The adopted software environment, combining Xilinx Vivado 2017.2 and LabVIEW 2019 myRIO Toolkit, provided a stable and compatible framework for integrating the XADC IP core into the NI myRIO-1900 platform. This compatibility simplified the deployment process and enabled continuous access to on-chip thermal measurements in real operating conditions.

The experimental thermal acquisition campaign successfully generated representative thermal signatures under the different FPGA operating scenarios introduced in the previous sections. The collected raw thermal dataset clearly exhibited distinct transient and steady-state behaviors, while the abnormal TSCA-like scenarios produced measurable deviations from the nominal thermal evolution. These observations confirm that thermal behavior can be used as an effective side-channel indicator for monitoring FPGA operational states.

The proposed thermal feature engineering strategy further enhanced the informational content of the dataset. In addition to the raw thermal measurements, differential features, statistical descriptors, sliding-window averages, and thermal slope indicators were extracted to capture both global and local thermal dynamics. The resulting feature space provided a richer representation of the physical system and supplied meaningful inputs for the machine learning stage.

Using this engineered dataset, the Isolation Forest algorithm demonstrated a satisfactory capability for separating normal and abnormal thermal behaviors, achieving an AUC greater than 0.70. Although the obtained performance does not represent the final objective of the proposed Digital Twin, it validates the overall methodology and confirms that the selected thermal features contain sufficient discriminative information for anomaly detection.

Although the experimental validation was conducted on a single NI myRIO-1900 platform embedding a Xilinx Zynq-7010 FPGA-SoC, the proposed methodology remains architecture-independent at the thermal behavioral level. Since the framework relies on thermal feature extraction and anomaly pattern modeling rather than device-specific logic structures, it can be extended to other FPGA-SoC and MPSoC architectures equipped with embedded thermal sensing capabilities.

Several research directions can further improve the proposed framework. First, extending the thermal feature engineering process by introducing additional temporal, frequency-domain, and correlation-based descriptors is expected to increase the separability between normal and abnormal operating conditions and improve the overall prediction accuracy. A larger and more diverse dataset covering additional FPGA workloads and environmental conditions would also strengthen the model generalization capability.

Future work will also investigate the integration of advanced machine learning and deep learning architectures, including Autoencoders, Long Short-Term Memory (LSTM) networks, and Transformer-based temporal models, to better capture complex thermal dependencies. Another promising direction consists of developing an adaptive online Digital Twin capable of continuously updating its model parameters from newly acquired thermal data, enabling real-time self-learning and long-term monitoring.

Given the lightweight nature of the proposed thermal feature extraction pipeline and the relatively low inference complexity of the Isolation Forest model, the framework remains suitable for future embedded Edge-AI deployment. Modern FPGA-SoC architectures, including recent Xilinx Zynq UltraScale+ MPSoC and Versal AI Edge platforms, integrate dedicated processing systems, programmable logic, on-chip memory resources, and AI acceleration engines capable of supporting real-time inference tasks. These heterogeneous architectures provide sufficient computational space to host thermal sensing, feature extraction, and anomaly inference directly on-device. In such a deployment, the FPGA could act as a self monitoring and self protecting thermal Digital Twin node, enabling autonomous online anomaly detection and dynamic reconfiguration or recovery mechanisms without external processing dependency.

Finally, the proposed methodology can be extended toward a complete cyber-physical security framework by combining thermal side-channel information with other embedded sensors and operational indicators. Such a multimodal Digital Twin could provide enhanced resilience against emerging hardware attacks and support predictive maintenance strategies for next generation FPGA-based cyber-physical systems.

## 6. Conclusions

This work presented an AI-enabled Thermal Digital Twin architecture for FPGA-SoC thermal monitoring and TSCA-like anomaly detection based on real-time on-chip thermal sensing, thermal feature engineering, and unsupervised machine learning. By exploiting the embedded XADC sensing infrastructure of the NI myRIO-1900 platform, representative thermal signatures were acquired under multiple operational and abnormal workload scenarios, enabling the construction of a structured thermal dataset for behavioral modeling.

The developed Thermal Digital Twin architecture demonstrated that engineered thermal descriptors, including statistical, differential, and temporal features, provide sufficient discriminative information for anomaly detection using an Isolation Forest model. Compared to conventional thermal monitoring approaches based on static thresholds or external sensing infrastructures, the proposed architecture offers several practical strengths, including direct on-chip thermal observability, lightweight anomaly inference, modular integration, and compatibility with embedded cyber-physical systems.

However, the current implementation remains limited by its offline analytical nature, validation on a single FPGA-SoC platform, and the use of a single embedded XADC junction sensor, which provides only global thermal observability without spatial thermal distribution across the FPGA fabric. In addition, the high-level synthesis abstraction of the NI LabVIEW FPGA environment limits deeper low-level architectural and placement-aware thermal exploration compared to manual HDL implementations.

Future work will focus on extending the thermal feature engineering space, integrating distributed thermal sensing infrastructures for finer spatial observability, diversifying FPGA architectural implementations and floorplanning strategies, validating the framework across multiple FPGA platforms under broader metrological conditions, and migrating the architecture toward online, real-time, and on-device Edge-AI deployment for autonomous thermal anomaly detection and adaptive cyber-physical protection.

## Figures and Tables

**Table 1:** Defined operating scenarios.

Scenario	Active Motors	TSCA-like Workload
S0-N	0	No
S0-A	0	Yes
S1-N	1	No
S1-A	1	Yes
S2-N	2	No
S2-A	2	Yes
S3-N	3	No
S3-A	3	Yes

**Table 2:** Summary of the constructed thermal dataset.

Dataset Component	Description
Total thermal windows	536
Normal thermal windows	268
TSCA-like anomalous windows	268
Number of thermal features	17
Raw thermal samples per window	60
Sampling interval	10 s
Window duration	10 min
Operating scenarios	S0, S1, S2, S3
TSCA-like scenarios	S0-A, S1-A, S2-A, S3-A

**Table 3:** Summary of Isolation Forest performance metrics.

Accuracy	Precision	Recall	F1-Score	FPR	FNR	AUC
75.2%	75.1%	75.4%	75.2%	25.0%	24.6%	0.762

## Data Availability

The raw thermal datasets and the engineered features generated during this study are part of an ongoing research effort on AI-enabled thermal digital twins and are not publicly available at this stage. They are available from the corresponding author upon reasonable request.

## Abbreviations

3D-IC	Three-Dimensional Integrated Circuit
ADC	Analog-to-Digital Converter
AI	Artificial Intelligence
AUC	Area Under the ROC Curve
ARM	Advanced RISC Machine
CMC	Canadian Microelectronics Corporation
CPS	Cyber-Physical System
DT	Digital Twin
DSP	Digital Signal Processing
FN	False Negative
FNR	False Negative Rate
FP	False Positive
FPR	False Positive Rate
FPGA	Field-Programmable Gate Array
FPGA-SoC	Field-Programmable Gate Array System-on-Chip
ICS	Industrial Control System
IIoT	Industrial Internet of Things
ILA	Integrated Logic Analyzer
IP	Intellectual Property
I/O	Input/Output
LIMA	Laboratoire d’Ingénierie et de Microsystèmes Avancés
ML	Machine Learning
MPSoC	Multiprocessor System-on-Chip
NI	National Instruments
PWM	Pulse Width Modulation
RAM	Random Access Memory
ROC	Receiver Operating Characteristic
SCADA	Supervisory Control and Data Acquisition
SiP	System-in-Package
SoC	System-on-Chip
SVM	Support Vector Machine
SYSMON	System Monitor
TDT	Thermal Digital Twin
TN	True Negative
TP	True Positive
TSCA	Thermal Side-Channel Attack
TSCA-like	Thermal Side-Channel Attack-like
UQO	Université du Québec en Outaouais
VI	Virtual Instrument
XADC	Xilinx Analog-to-Digital Converter
